# Primary Intracranial Leiomyosarcoma Secondary to Glioblastoma: Case Report and Literature Review

**DOI:** 10.3389/fonc.2021.642683

**Published:** 2021-05-20

**Authors:** Liyan Zhao, Yining Jiang, Yubo Wang, Yang Bai, Ying Sun, Yunqian Li

**Affiliations:** ^1^ Department of Clinical Laboratory, Second Hospital of Jilin University, Changchun, China; ^2^ Department of Neurosurgery, First Hospital of Jilin University, Changchun, China

**Keywords:** primary intracranial leiomyosarcoma, glioblastoma, leiomyosarcoma, treatment, prognosis, genetic diagnosis

## Abstract

**Background:**

Leiomyosarcoma is a highly malignant soft-tissue sarcoma with a poor prognosis. In recent years, treatment for leiomyosarcoma has not shown much progress. Primary intracranial leiomyosarcoma (PILMS) is a much rarer type of neoplasm, which occurs more frequently in immunocompromised patients. PILMS cases reported in the literature are scarce and treatment strategy and prognosis are still under debate. In this study, a case of PILMS secondary to the total resection of giant cell glioblastoma is reported.

**Case Description:**

A 38-year-old male was hospitalized with a three-month history of a temporal opisthotic bump. His medical history included a total resection of a tumor located in the right temporal lobe performed 4 years earlier. Pathological examination led to a diagnosis of giant cell glioblastoma, and the patient underwent postoperative chemotherapy with temozolomide for 6 weeks plus simultaneous radiotherapy with 63.66 Gary. Four years later, during regular follow-up, a preoperative MRI brain scan resulted in a well-defined signal pointing out two nodule-like features located at the right temporal lobe and subcutaneous soft tissue, respectively, and near the area where the previous giant cell glioblastoma was located. The mass was completely removed by a transtemporal approach and postoperative pathology revealed that the mass was a leiomyosarcoma. The patient underwent postoperative radiotherapy and no recurrence occurred until now.

**Conclusions:**

To date, research on soft-tissue sarcoma, especially PILMS, has not made much progress, and a limited number of studies have provided few details on the management of PILMS. The treatment of choice for PILMS is aggressive multimodal treatment based on total tumor resection and radiotherapy. Moreover, systemic treatment with chemotherapy and targeted therapy, such as olaratumab, as well as further research still needs to be performed as many questions are left unanswered. To our knowledge, this is the first report on a case of PILMS secondary to glioblastoma, which might serve as a potential reference for clinicians and clinical studies.

## Introduction

Intracranial leiomyosarcoma (LMS) is rare, and most often occurs as a result of metastasis of primary smooth muscle tissue tumors that can therefore develop in different organs due to the ubiquitous presence of the smooth muscle tissue in the body (1–4). Primary intracranial leiomyosarcoma (PILMS) is extremely rare in the central nervous system (CNS) and previous studies suggested that less than 1% of brain biopsies (or 3 out of 25,000 brain tumors) are positive for LMS (5). They are speculated to derive from smooth muscle cells of the blood vessels or dura mater pluripotent mesenchymal cells (1, 2, 6–9), and display strong smooth muscle differentiation (10). In addition, PILMS usually occurs in immunocompromised patients or after exposure to radiation (8, 11, 12). Here we report a case of PILMS arising on the right temporal robe near to the location of a previous giant cell glioblastoma (GCG) totally excised 4-year earlier. The patient was not immunocompromised. To the best of our knowledge, this is the first report describing a case of PILMS secondary to a glioblastoma. Relevant literature has been reviewed, and diagnosis, and prognosis, especially regarding treatment strategy have been discussed.

## Case Report

### History and Examination

**Figure 1:**
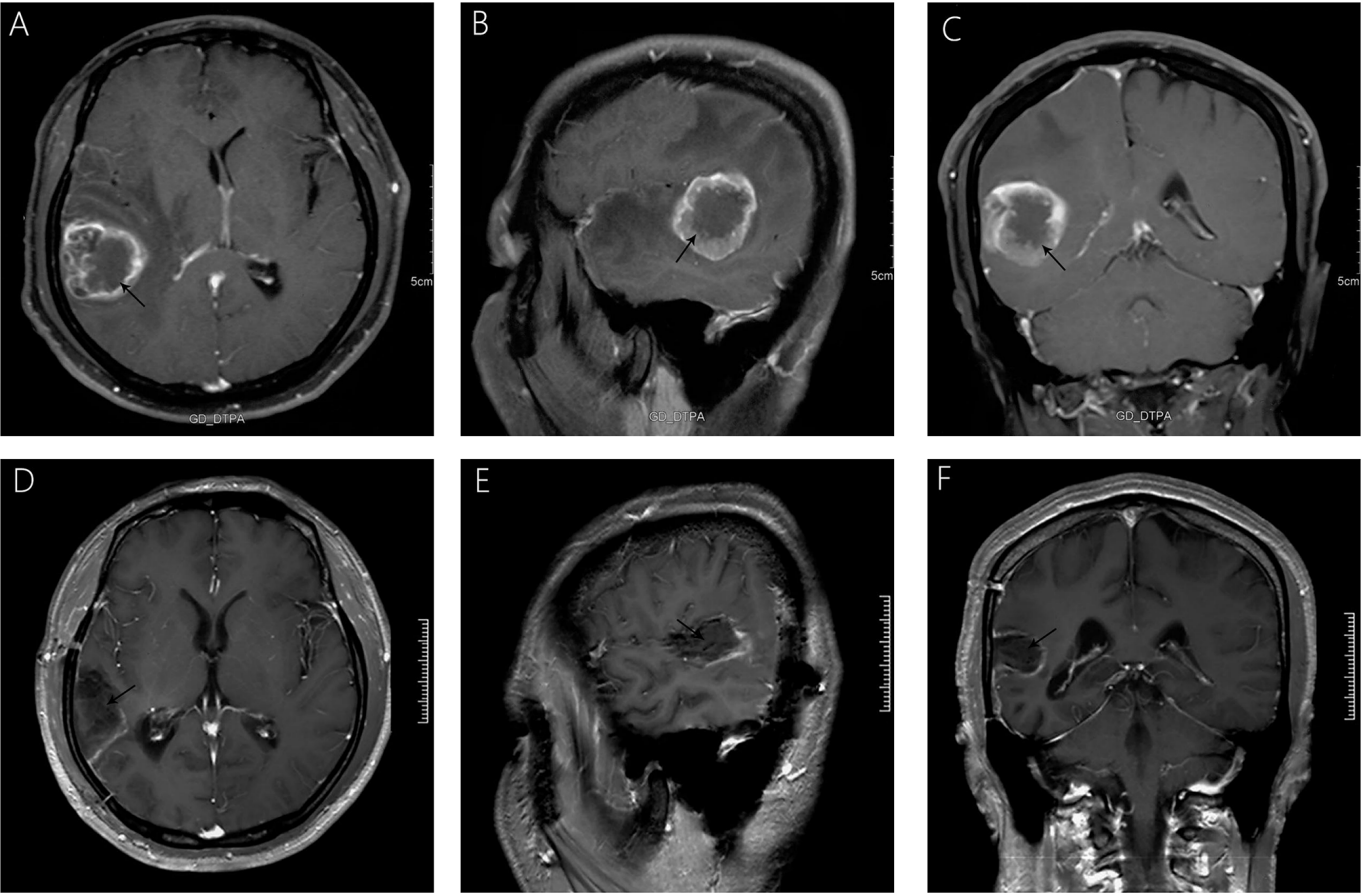
Significant heterogeneous enhancement is observed with evident edema after gadolinium administration **(A–C)**. A follow-up MRI, 3 months after surgery **(D–F),** showed that the lesion was completely removed, without any signs of recurrence.

**Figure 2:**
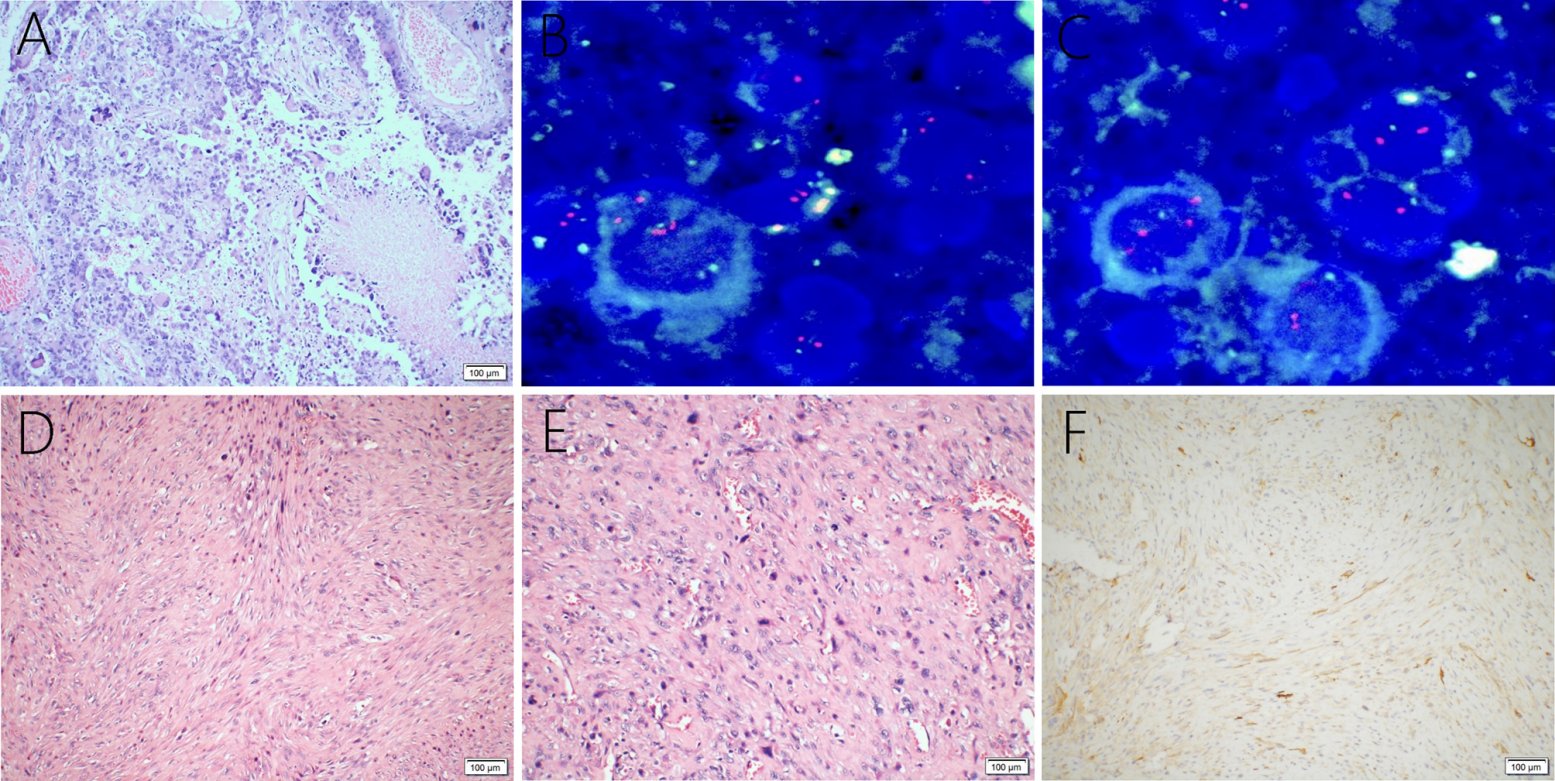
Giant cell glioblastoma is composed of large, closely-arranged cells, with an eosinophilic cytoplasm and obvious nuclear atypia. There are also scattered multinucleated giant cells. Local necrosis and vascular proliferation are observed **(A)**. FISH detection suggests no loss of heterozygosity in *1p* **(B)** or *19q* **(C)** chromosomes. Primary intracranial leiomyosarcoma showing spindle−shaped cells **(D)** and abundant mitotic activity **(E)** through the tumor, hematoxylin, and eosin staining. Immunohistochemical examination was positive for H-caldesmon **(F)**.

**Figure 3:**
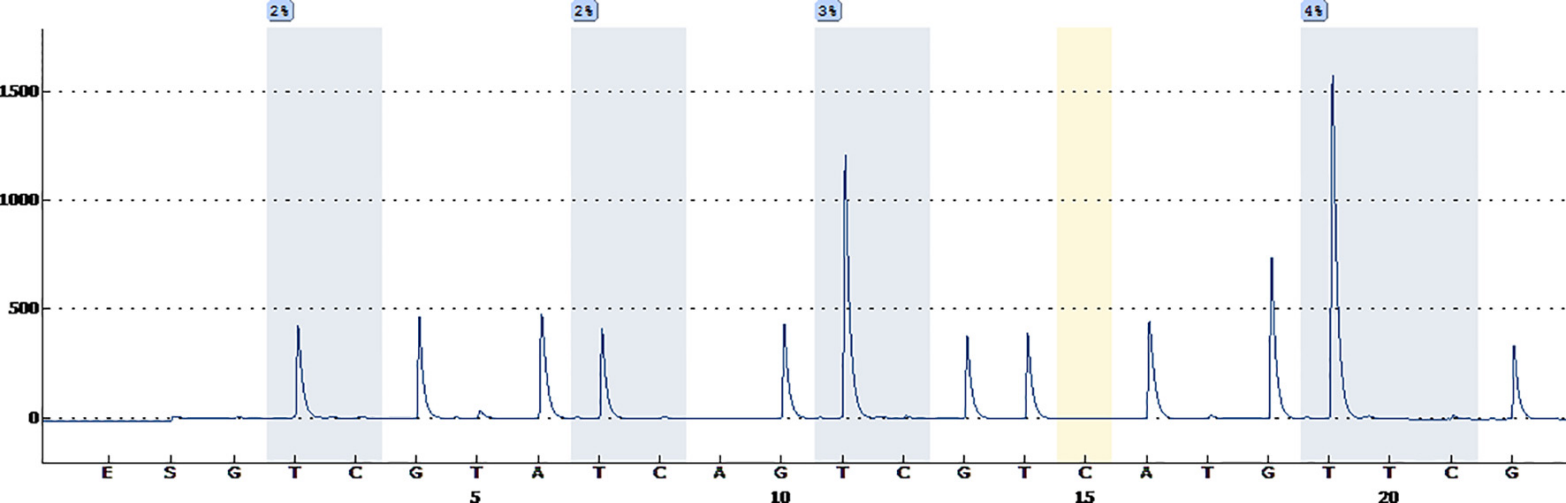
Pyrosequencing demonstrates that no O6-methylguanine-DNA methyltransferase promoter methylation was found.

**Figure 4:**
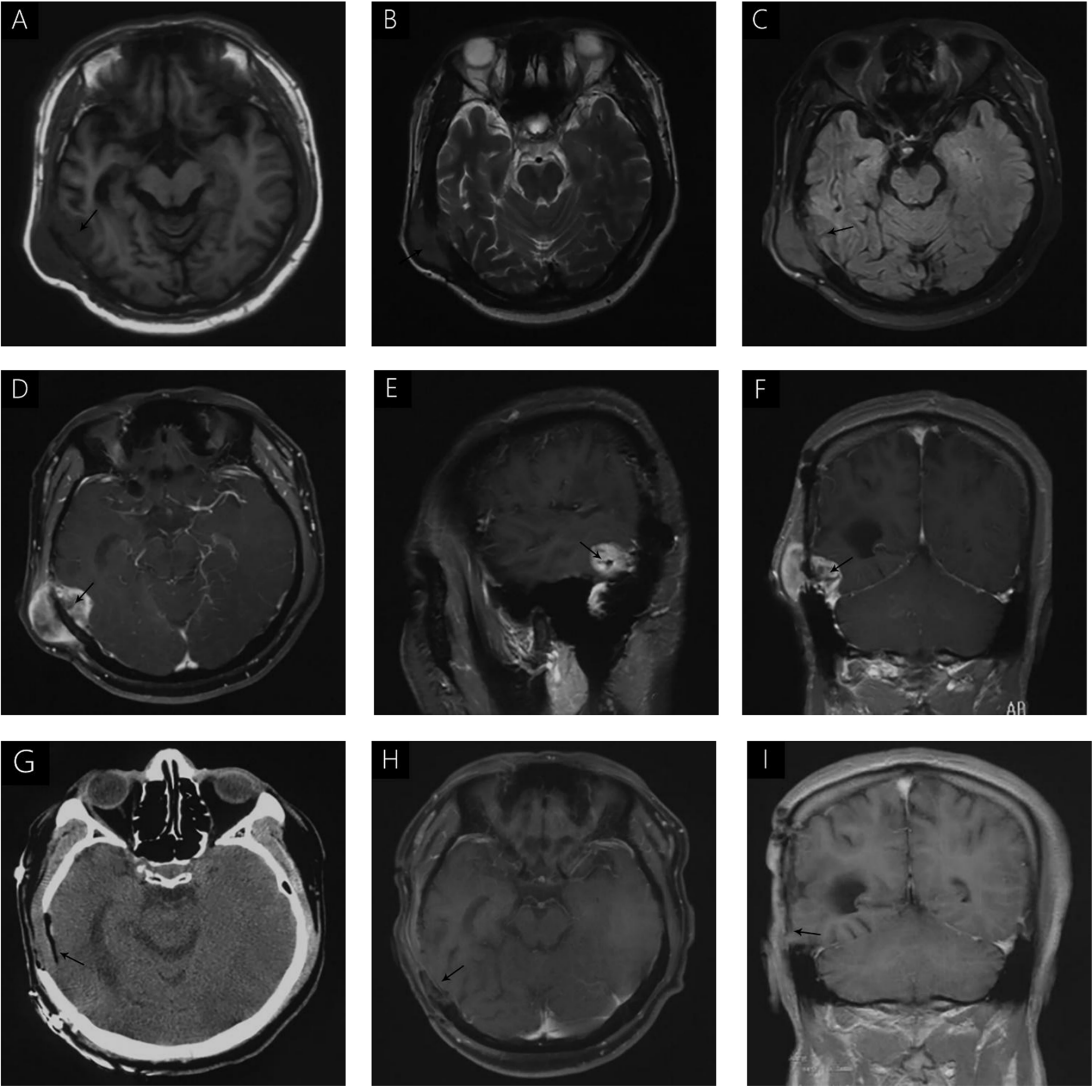
**(A)** hypointense signal is seen on T1WI **(A)**. Isointense and slightly hyperintense signals were seen on T2WI **(B)**; Isointense signal on FLAIR **(C)**. Significant enhancement was seen after gadolinium administration, without uniform enhancement in the center of the lesion **(D–F)**. Immediate postoperative CT **(G)** and follow-up MRI, 3 months after surgery **(H, I)** demonstrated complete removal of the tumor and no signs of recurrence.

A 38-year-old male with a 3-month history of a temporal opisthotic bump was admitted to the hospital. The patient had no history of immunosuppressive medical treatment, intravenous drug use, previous organ transplantation or sexual promiscuity. Moreover, he did not experience any signs of headaches, dizziness, nausea, vomiting nor did he have any other sensory or motor deficits. Routine laboratory analysis showed standard parameters within normal limits, and the serologic test was negative for Human Immunodeficiency Virus (HIV), Hepatitis-B Virus (HBV), Hepatitis-C Virus (HCV), and Epstein Barr virus (EBV). According to his medical history, the patient underwent craniotomy 4 years earlier, because of the presence of an abnormal and heterogeneous magnetic resonance imaging (MRI) enhancement signal located in the right temporal and parietal lobe, together with evident edema (**Figures 1A–C**). The MRI performed 3 months after follow-up (**Figures 1D–F**) revealed that the tumor was successfully removed. Postoperative pathological examination of the tumor led to a diagnosis of GCG, with a Ki-67 index of 50%. Histological examination showed that many giant tumor cells were densely arranged, with blood vessel hyperplasia and focal necrosis. The tumor cells had obvious atypia and eosinophilic cytoplasm, and the nuclei were eccentric with mitosis that was easy to observe (**Figure 2A**). Moreover, pathological findings were evaluated. Pyrosequencing presented that no O6-methylguanine-DNA methyltransferase promoter methylation was shown (**Figure 3**). Fluorescence *in situ* hybridization suggested no loss of heterozygosity in 1p/19q chromosome (**Figures 2B, C**). Levels of *IDH1*, *TERT*, and *BRAF* were determined by multiple polymerase chain reaction amplification combined with high-throughput sequencing, which did not indicate a mutation of *IDH1-R132/R172*, *TERT-C228T/C250T* or *BRAF-V600E*. The patient accepted to be subjected to postoperative chemotherapy with temozolomide for 6 weeks, and concomitant local 63.66 Gray radiotherapy. Follow-up was not stopped after the first surgery. During a visit after 4 years when the patient presented the bump, the MRI brain scan resulted in a well-defined signal pointing out two nodule-like features of 3.1x2.5 cm and 4.0x1.8x3.7 cm located kin the right temporal lobe and subcutaneous soft tissue, respectively (**Figures 4A–F**), with a slightly hypointense signal on T1-weighted imaging (T1WI, **Figure 4A**), isointense and slightly hyperintense signal on T2-weighted imaging (T2WI, **Figure 4B**), and isointense signal on fluid attenuated inversion recovery (**Figure 4C**). The lesion showed significant edge enhancement and heterogeneous reinforcement inside the tumor (**Figures 4D–F**). A preoperative diagnosis of a recurrent glioblastoma was made.

### Surgery

The patient underwent transtemporal craniotomy under preoperative and intraoperative neuronavigation, together with electrophysiological monitoring. The tumor was pinkish in color, solid, bloody, and was closely adherent to the brain parenchyma and subcutaneous tissue. The entire tumor was removed.

### Histopathological Findings

The postoperative histopathological examination led to a diagnosis of leiomyosarcoma. The microscopic examination revealed the tumor was composed of spindle-shaped cells (**Figure 2D**) with an abundant mitotic activity (**Figure 2E**), and the Ki-67 labeling index was 10-20%. Immunohistochemical staining was positive for h-caldesmon (**Figure 2F**), vimentin, SMA, and desmin, but negative for S-100, epithelial membrane antigen (EMA), glial fibrillary acidic protein (GFAP), CK-pan, CD34, CD31, CD117, Oligo-2, Dog-1, PR, and STAT6.

#### Postoperative Course

The postoperative course was uneventful and no postoperative complications occurred. In addition, positron emission tomography (PET) was performed to identify potential extracranial primary sites, and serum tumor markers were measured; both were negative. One month after surgery, the patient underwent postoperative 54 Gray radiotherapy. Immediate post-operative cranial CT (**Figure 4G**) and follow-up MRI were performed 3 months after surgery (**Figures 4H, I**), and demonstrated complete removal of the tumor and no signs of recurrence. During the last telephone follow-up in December 2020, the patient stated that he did not report any abnormal condition and that he was leading a normal daily life. Based on these results, his conditions seem stable and therefore, the patients will undergo routine follow-up with MRI.

## Discussion

### Epidemiology

LMS is an uncommon malignant tumor, accounting for 1% of the head and neck soft-tissue sarcomas (STS) (13), being usually the result of metastasis of primary smooth muscle tissue tumors (1–4, 14). PILMS is rare and sporadically reported (4, 14), indeed accounting for approximately 0.1% of all the intracranial tumors (3). The onset range is quite wide, from 4 to 75 years of age (2, 15, 16), commonly appearing in the second and third decades of life (1, 6, 17–19), and in previous studies, a slight male predominance was observed (2, 20).

### Etiology

The cellular origin and tumorigenesis of PILMS is considered as deriving from the smooth muscle cells of the blood vessels or the pluripotent mesenchymal stem cells in the dura mater (1, 2, 6–9). LMS also mostly occur in immunocompromised patients compared to the immunocompetent ones (11), especially patients with HIV (7, 12, 17, 21, 22) or EBV (1, 5, 12, 21, 23, 24) infection, subjected to organ transplantation (18), with malignancies (20), genetic disorders (18), after radiation exposure (25), and especially in children (8). Therefore, immunodeficiency plays a role in the occurrence and development of PILMS.

Co-infection with EBV in immunosuppressed patients with HIV is considered a leading factor in the development of LMS (9, 22, 26, 27), and EBV-transformed and infected smooth muscle cells may contribute to the pathogenesis of LMS in patients with AIDS (2, 3, 17). However, immunocompetent patients in whom LMS occurred, were invariably negative for EBV (2, 17, 24, 28). Radiotherapy (2) and chemotherapy (27, 29) are also considered as potential factors inducing PILMS. Radiation oncogenesis was first defined by Cahan et al. (30), in 1948. Since then, it was clear that radiation doses above 50 Gray cause cell death, while lower doses (e.g.< 30 Gray) are associated with genomic instability and cell repair mechanisms of the caused damages (2). Suzuki et al. (31), also described a radiation-induced sarcoma usually within or at the edge of the tumor. Since the radiation is not uniformly distributed within the tissue, a sufficient dose does not reach the edge to ensure the killing of all tumor cells (31). Furthermore, Fujimoto et al. (9), described a case of LMS arising after the resection of a neurofibroma at the left cerebellopontine angle and concluded that the mechanical and/or heat stimulation during a previous surgery was associated with the development of LMS. The patient in this case report is the first ever described with PILMS secondary to a glioblastoma, and the pathogenesis was hypothesized as associated with the history of malignant tumor and radiation exposure together with the mechanical and heat stimulation during the first surgery. Nevertheless, these hypotheses did not explain all cases of PILMS in immunocompetent patients and further studies are needed to clarify the clinical features involved.

### Clinical Presentation

No specific symptoms are shown in patients with PILMS, and they largely depend on the location of the tumor and the mass effect (3, 4, 16, 32); the average length of the symptoms approximately 4 months (3, 33). General symptoms include headache (1, 32), memory impairment (17), gait instability (1), altered mental status (34), and seizures (1). In addition, some patients may present subdural hematoma (3, 20) or intratumoral apoplexy (15, 20). The subdural hematoma is hypothesized to be developed from new capillary formation, vascular hyperpermeability, and serum protein exudation, as a result of a typical inflammatory reactions (3).

### Radiological Characteristic

Due to the rarity of PILMS, radiological details describing it are lacking in the literature. In general, PILMS presents a hyperdense signal on CT and homogenous enhancement on enhanced scanning images (3, 16). In addition, calcifications can be observed (16, 35). MRI is considered the primary neurological approach to assess PILMS, which is also important for surgical planning (14). PILMS can develop as either extra-axial or intra-axial tumor, and the imaging features are different in these two locations (4). The extra-axial PILMS is usually characterized by uniform hypointense or isointense T1WI and T2WI (4, 36). After gadolinium enhancement, well-defined homogeneous (22, 23, 28) or inhomogeneous (17, 37) enhancement with (6) or without (4) dural tail signal is detected, resembling meningioma (18, 22, 23, 28, 38). Moreover, the tumors were always significantly enhanced (18, 36). No significant differences in survival were observed in patients with dura involvement (39). Intra-axial PILMS often appears as an irregular mass presenting a heterogeneous intense and heterogeneous enhanced pattern (2, 24, 26, 40). Ultra-sound examination could also be performed for patients with skull involvement (36).

### Diagnosis

The diagnosis and differential diagnosis of PILMS depend on the comprehensive analysis of all the laboratory tests, MRI and pathological examination (2, 5, 6, 9). Histological examination revealed the presence of elongated spindle-shaped cells with pleomorphism and coagulative necrosis (17, 41), and most of these cells grow following a fascicular pattern (6, 16, 36). Moreover, PILMS is often positive for desmin, actin, h-caldesmon, α-SMA, and occasionally vimentin (6, 9, 16, 28), and is negative in S-100, EMA and GFAP (6, 9, 16, 28). In addition, PILMS showed a high Ki-67 index and mitotic index (16). H-caldesmon is considered a specific and valuable biomarker characterizing smooth muscle cells and LMS (9, 24). Extra-axial PILMS is sometimes difficult to differentiate from meningioma on radiological examination (6, 9, 24, 34). However, EMA is immunohistochemically positive in meningioma, meningosarcoma and hemangiopericytomas (9, 24), thereby helping in the differential diagnosis. Notably, thorough investigation, including whole-body CT, bone scans, lumbar punctures, and PET scans were imperative to exclude extracranial lesions.

### Treatment

Due to the rarity of PILMS, standard management guidelines have not yet been established (39). However, currently, a multimodal approach, including surgery, radiotherapy, and chemotherapy is the main treatment (12, 21, 42). In addition, surgical resection is the leading treatment to perform gross tumor resection (GTR) and the achievement of negative surgical margins due to extension of the resection is one of the most frequently reported predictors of recurrence and survival (2, 15, 16, 28, 33). Zhang et al. (39) demonstrated that the extent of excision might result in differences because of the difference in score systems that are presented in the literature, which needs further unification.

### Radiotherapy

In PILMS, postoperative radiotherapy is used to control local recurrence (2, 43). In many previous studies, GTR combined with postoperative radiotherapy is indeed the main treatment strategy for PILMS (1, 3, 26, 27, 44, 45). However, the specific benefit of radiotherapy in terms of the survival of PILMS patients is not clear (39). To date, there is no consensus that patients with PILMS should undergo radiotherapy regardless of the extent of resection. In several studies, radiotherapy was not recommended for patients with GTR (15). However, considering the aggressive character, immediate adjuvant radiotherapy after GTR was approved in some cases (3, 28, 37). In this case, the patient underwent radiotherapy immediately after GTR, and had a relatively good survival without recurrence. In addition, radiation therapy represents an adjuvant option in patients with relapse or progression (39). In case of recurrent LMS, Gallagher et al. (28), suggested to perform a re-irradiation according to their experience in the treatment of recurrent glioblastoma. Recently, gamma knife radiosurgery (43, 46) and stereotactic robotic cyber knife radiosurgery (47) have been performed to treat PILMS, revealing their feasibility and effectiveness in treating this type of tumor, although the number of patients was small, thus, they can be considered potential treatment strategies. The specific treatment advantage needs to be verified in future multicenter prospective studies.

### Chemotherapy

In previous studies, it was demonstrated that PILMS is inclined to progress to extra-cranial metastasis, such as the spinal cord, lung, pleural, spleen, and hip (6, 26, 48). Although the role of chemotherapy in preventing extracranial metastasis is currently unknown, we speculate that it is difficult for radiation alone to confine the aggressive behavior of LMS. Chemotherapy is not a routine treatment of PILMS (9, 17), and the choice of effective chemotherapeutic agents remains unclear (28). Because of its good blood brain barrier permeability (BBBP) and acceptable level of toxicity, temozolomide was the first chemotherapy drug used in the treatment of PILMS (49). Temozolomide has moderate activity in residual or metastatic STS, with a response rate of 8% (41, 49, 50), which makes it a promising drug in the treatment of PILMS. In some studies, it was revealed that temozolomide was effective at a dose that was equivalent to that of dacarbazine (9, 51, 52). However, the validity of monotherapy of chemotherapeutic drugs has been questioned (3), and the combination with other therapeutic approaches seems somewhat effective (1–3, 6). In a recent study, Francisco et al. (53), reported a case of PILMS where maintenance treatment involved temozolomide and nimotuzumab. Nimotuzumab is an epidermal growth factor receptor monoclonal antibody. STS, like LMS, can express epidermal growth factor receptor-34, and its blocking promoted tumor inactivation and decreased chemoresistance (54). However, in this study, no improvement in survival was observed. For the current treatment of sarcomas, anthracyclines (doxorubicin and epirubicin) remain the first-line standard treatment regimens of advanced STSs (55), with a median overall survival (OS) of 12-18 months (56, 57). When combined with other drugs, such as ifosfamide, a significant improvement in the response rate and progression free survival (PFS), but not in OS, was observed (58, 59). The treatment experience with anthracyclines in PILMS is limited and controversial, and no improvement in survival was observed (60, 61), due to its poor BBBP and limited treatment experience of this disease (12, 42).

### Targeted Therapy

Here, we describe the application and research progress of targeted drugs in PILMS. To date, monoclonal antibodies to LMS, especially to PILMS, are scarce (28). However, along with increasing the understanding of the pathophysiology and underlying molecular mechanisms of action of LMS, there is an increase in research studies. For example, somatostatin receptor subtypes have been detected in moderate and malignant sarcomas. In 2016, Crespo-Jara A et al. (62), reported a case of metastatic and multiple drug-resistant sarcoma, which was successfully treated with radiolabeled somatostatin analogs. However, this agent has not been approved in China. Moreover, platelet-derived growth factor receptor (PDGFR), especially the alpha (α) isoform, has been confirmed to be associated with the metastasis and proliferation of LMS cells. Therefore, drugs that block the action of PDGFR could be a promising antitumor regimen (63, 64). Lartruvo^®^ (olaratumab) is a PDGFR-α antagonist, a first-in-class recombinant human immunoglobulin-G subclass-1 monoclonal antibody that blocks binding and activation of the PDGF receptor (55). In 2016, a Phase 1b study and randomized Phase II clinical trials, showed that advanced STS treated with olaratumab plus doxorubicin had a significant prolonged OS when compared to doxorubicin monotherapy (26.5 *vs* 14.7 months; HR 0.46, 95% CI 0.30-0.71, p=0.0003), and slight PFS extension (6.6 *vs* 4.1 months; HR 0.672, 95% CI 0.442-1.021, p=0.0615) (65), with manageable toxicity. This was the first randomized trial showing a significant improvement in OS, compared to doxorubicin monotherapy, which brings light for advanced STS and PILMS patients. From 2016 to 2019, olaratumab was considered the most effective PDGFRα neutralizing antibody for LMS (56). However, the latest Phase 3 multicenter randomized clinical trials in 2020 failed to demonstrate OS benefits of doxorubicin plus olaratumab when compared to doxorubicin plus placebo in advanced STS (20.4 *vs* 19.7 months; HR 1.05, 95% CI 0.84-1.30, p=0.69) and LMS (21.6 months *vs* 21.9 months; HR 0.95, 95% CI 0.69-1.31, p=0.76) (66). The reason for the differences between the results of the second and third clinical trial has not yet been identified. Gennatas et al. (67), reviewed eight patients with advanced STS who underwent at least two treatment cycles of doxorubicin plus olaratumab between May 2017 and March 2019, and none of the patients experienced survival benefits. At present, the treatment of STS with olaratumab has been suspended by the drug manufacturer of olaratumab (66), and further studies are needed. Moreover, no studies have been performed with olaratumab for PILMS or its BBBP. Other recent first-line studies of advanced STS included the comparison of doxorubicin monotherapy with docetaxel plus gemcitabine (58) or doxorubicin plus ifosfamide (51, 59), palifosfamide (68), or evofosfamide (65). However, historical OS (12-18 months) and the 2-year survival rate (20-30%) did not show improvement. Thus, identifying new and effective treatment for advanced STS, especially PILMS, is of utmost importance. Mathieson et al. (3), described a treatment including the combination of vincristine, ifosfamide, doxorubicin, and etoposide with radiotherapy on a pediatric PILMS patient, which did not result in recurrence in 18 months. The use of the vascular endothelial growth factor inhibitor bevacizumab is also a promising approach, and has been increasingly used to treat LMS (32, 52). Three Phase II clinical trials demonstrated that bevacizumab is an effective treatment for some STS (69, 70). Gallagher et al. (28), also reported recurrence of PILMS treated with re-operation and bevacizumab (7.5mg/kg, 4 doses at 3-week intervals), and the follow-up was lost two-months after the re-operation. Notably, active antiretroviral therapy is imperative for PILMS patients with retroviral infections, such as HIV (28).

### Prognosis

The prognosis of PILMS is overall poor (3, 4, 15, 32, 33), and the long-term prognosis is not clear (53). A limited number of studies reported a survival range from 6 to 44 months (4, 37, 45), and the average follow-up was 12 months, although Niwa et al. (19 *)*, reported a patient who died 8 years after the initial surgery, which represented the longest survival ever published. The poor prognosis and high local recurrence rate were considered as associated with the difficulty in obtaining negative surgical resection margins and an inadequate radiotherapeutic dose (6, 14, 28). Vos et al. (71), contrasted the survival period of patients with STS between 2010-2014 and 1989-1994, and found that the OS had improved, but was not statistically significant. In some studies, it was shown that gender, age, immunosuppressive status, dural origin, or EBV infection did not have a significant impact on treatment outcome, but tumor size, location, mitotic rate, residual tumor, and inadequate dose of radiation were all unfavorable factors with a negative effect on survival (2, 15, 16, 72). However, Zhang, et al. (39), demonstrated that age, tumor size, and location were not statistically linked with clinical outcome. Shotton et al. (73), also suggested that the perineural invasion is an important predictor of survival and recurrence (10). The local recurrence rate is approximately 25.9% after radiotherapy on the initial lesion (15). Taken together, GTR has a significant and unexpected favorable outcome on survival (17, 24, 39).

## Conclusion

Since PILMS is an extremely rare type of neoplasm, studies reporting on PILMS cases are rare. More future clinical trials, treatment experience, and long-term follow-up are required to fully understand this disease. Olaratumab might be a potential targeted drug for the treatment of PILMS, but has never been applied to PILMS patients. Thus, further studies are needed for its validity and BBBP. Here, we reported on PILMS secondary to GCG for the first time, and represented an additional reference among the few available, which might serve as a potential guide for clinicians and clinical studies.

## Ethics Statement

The studies involving human participant was reviewed and approved by Ethics Committee of the First Hospital of Jilin University. The patient/participant provided his written informed consent to participate in this study. Written informed consent was obtained from the individual(s) for the publication of any potentially identifiable images or data included in this article.

## Author Contributions

LZ and YJ made study design, data collection, data analysis and interpretation, and composed the manuscript and literature review. YL and YW were the surgeons that performed the surgery, and did data collection, data analysis, and interpretation. YS and YB made English and grammar corrections, critical revisions, and approved final version. YL had the acquisition, analysis or interpretation of data for the work, revising it critically for important intellectual content, final approval of the version to be published, and agreed to be accountable for all aspects of the work in ensuring that questions related to the accuracy or integrity of any part of the work are appropriately investigated and resolved. All authors contributed to the article and approved the submitted version.

## Conflict of Interest

The authors declare that the research was conducted in the absence of any commercial or financial relationships that could be construed as a potential conflict of interest.

## Abbreviations

CT, computed tomography; LMS, leiomyosarcoma; PILMS, primary intracranial leiomyosarcoma; STS, soft-tissue sarcomas; GCG, giant cell glioblastoma; IDH, isocitrate dehydrogenase; MGMT, O6-methylguanine-DNA methyltransferase; EMA, epithelial membrane antigen; GFAP, glial fibrillary acidic protein; HIV, human immunodeficiency virus; HBV, hepatitis-B virus; HCV, hepatitis-C virus; EBV, Epstein-Barr virus; MRI, magnetic resonance imaging; PET, positron emission tomography; CNS, central nervous system; T1WI, T1-weighted imaging; T2WI, T2-weighted imaging; BBBP, blood brain barrier permeability; PFS, progression-free survival; OS, overall survival; GTR, gross tumor resection; PDGFR, platelet-derived growth factor receptor.
